# Deciphering the Immune–Tumor Interplay During Early-Stage Lung Cancer Development *via* Single-Cell Technology

**DOI:** 10.3389/fonc.2021.716042

**Published:** 2022-01-03

**Authors:** Wei-Wei Chen, Wei Liu, Yingze Li, Jun Wang, Yijiu Ren, Guangsuo Wang, Chang Chen, Hanjie Li

**Affiliations:** ^1^ Department of Clinical Oncology, University of Hong Kong, Hong Kong, Hong Kong SAR, China; ^2^ CAS Key Laboratory of Quantitative Engineering Biology, Shenzhen Institute of Synthetic Biology, Shenzhen Institutes of Advanced Technology, Chinese Academy of Sciences, Shenzhen, China; ^3^ Department of Thoracic Surgery, Shanghai Pulmonary Hospital, Tongji University School of Medicine, Shanghai, China; ^4^ Department of Thoracic Surgery, Shenzhen People’s Hospital, The Second Clinical Medical College, Jinan University, The First Affiliated Hospital, Southern University of Science and Technology, Shenzhen, China

**Keywords:** tumorigenesis, early-stage lung cancer, single-cell sequencing technology, immune-editing, immune evasion, tumor immunology

## Abstract

Lung cancer is the leading cause of cancer-related death worldwide. Cancer immunotherapy has shown great success in treating advanced-stage lung cancer but has yet been used to treat early-stage lung cancer, mostly due to lack of understanding of the tumor immune microenvironment in early-stage lung cancer. The immune system could both constrain and promote tumorigenesis in a process termed immune editing that can be divided into three phases, namely, elimination, equilibrium, and escape. Current understanding of the immune response toward tumor is mainly on the “escape” phase when the tumor is clinically detectable. The detailed mechanism by which tumor progenitor lesions was modulated by the immune system during early stage of lung cancer development remains elusive. The advent of single-cell sequencing technology enables tumor immunologists to address those fundamental questions. In this perspective, we will summarize our current understanding and big gaps about the immune response during early lung tumorigenesis. We will then present the state of the art of single-cell technology and then envision how single-cell technology could be used to address those questions. Advances in the understanding of the immune response and its dynamics during malignant transformation of pre-malignant lesion will shed light on how malignant cells interact with the immune system and evolve under immune selection. Such knowledge could then contribute to the development of precision and early intervention strategies toward lung malignancy.

## Introduction

As a life-threatening disease, lung cancer was estimated to cause more than 1.8 million deaths per year all over the world, with a 5-year survival rate of less than 20% (1). Based on the pathological type, lung cancer is divided into small cell lung cancer and non-small cell lung cancer (NSCLC), with the latter accounting for approximately 85% of the cases (1, 2). The histological subtypes of lung cancer are complex and highly various, which makes it challenging for the early diagnosis and treatment of lung cancer. Thus, accurate and comprehensive clinicopathological classification is critical in guiding the clinical treatment and predicting the prognosis of lung cancer (3–5). In 2016, the International Association for the Study of Lung Cancer (IASLC) proposed the 8th edition of TNM staging. Lung adenocarcinoma (LUAD) and its precursors range from atypical adenomatous hyperplasia, invasive adenocarcinoma *in situ*, micro-invasive adenocarcinoma, to eventually invasive lung adenocarcinoma (6). If lung cancer can be identified and treated at an early stage, before angiogenesis and invasion, patients have a greater chance of better disease control rate and survival rate.

Although many therapies, including chemotherapy, radiosurgery, targeted therapy, and immunotherapy, have been applied for lung cancer treatment, the 5-year survival rate is only 50% for patients with early-stage lung cancer (7). Surgery accounts for the primary first-line treatment for patients with early-stage lung cancer (8). However, patients with early-stage lung cancer may be diagnosed as multiple lesions, which occurs in 30%–50% of early-stage lung cancer (9). Besides, multiple lesions may occur simultaneously or successively in patients with lung cancer (10). There are great limitations in the radical and surgical treatment for multifocal early lung adenocarcinoma, while chemotherapy and targeted therapy cannot ameliorate the dilemmas, either. Thus, it is urgent to develop a novel therapy regimen for patients with early-stage lung cancer. With the recent development of tumor immunology, immunotherapy has provided new options for lung cancer patients (11–13). The emergence of immune checkpoint inhibitors has opened a new era of cancer therapy. Anti-PD-1/PD-L1 immunotherapy, an immune normalization therapy, selectively reinvigorates the anti-tumor immune responses in the tumor microenvironment (TME) with fewer immune-related adverse events (14, 15). Immunotherapy combined with surgery shows impressive clinical benefits in early-stage resectable NSCLC (16). Numerous ongoing clinical trials of immunotherapies and the novel combination therapies suggest that immunotherapies can be an optimal treatment strategy for unresectable early-stage NSCLC (14). However, the 5-year survival rate in NSCLC patients after combined surgery with immunotherapy treatment is still not ideal (17, 18). On the other hand, patients with the same TNM stage showed different prognosis outcomes after immunotherapy (19). Therefore, despite its success, it is still pressing to disentangle the complicated interactions between the immune system and tumor progression for developing novel and more effective strategies for the immune diagnosis and immunotherapies of lung cancer (20–22).

Profiling of the molecular states of all cell types within the lung tissue is currently revolutionizing the discovery of the mechanisms of lung cancer development (23) and can provide plentiful novel insights into the immune–tumor interplay in the early stage of lung cancer (24). The single-cell sequencing technologies in transcriptomics, genomics, epigenomics, proteomics, metabolomics, and spatial information have revolutionized biomedical research. The application of these tools enables the multidimensional study of organs, from cell atlas profiling, cell fate determination, cell–cell interaction to spatial construction (25, 26). Single-cell multi-omics have also emerged in recent years. All aspects of the cell, including a full history of its molecular states, spatial positions, and environmental interactions can be examined at the level of single cell by multimodal technologies and integrated computational methods (27). These methods demonstrated the power of simultaneously characterizing multiple levels of the immune response, which may boost our understanding of the underlying molecular mechanisms on how tumor evolves, and therefore contribute to the early detection and treatment of lung cancer by aiding the rational design of innovative diagnostic and personalized management approaches for patients (28, 29) (**Table 1**). Here, we discuss recent progress in employing multidimensional single-cell sequencing technologies to investigate the initiation and development processes of the pre-malignant lesion into lung cancer.

**Table 1:** Important discoveries of single-cell technology in the evolution of early-stage lung cancer.

Fields of Lung Cancer	Years	Pathological Type	Conclusions	Reference
Tumor Heterogeneity	2015	LUAD	Identified intratumoral and intertumoral heterogeneity and the correlation with prognosis	(30)
	2017	SCLC	Proposed a novel mutation profile and expression characteristics of SCLC	(31)
	2020	LUAD	Detected heterogeneity at the molecular level in each tumor and stromal cells of GGN more effectively	(32)
	2021	LUAD	Characterized the heterogenetic of tumor cells, immune cells, and stromal cells in SSN lesions	(33)
Evolution and Metastasis	2014	NSCLC	Detected the differential expression in metastasis-associated cancer initiation cells	(34)
	2020	LUAD	Revealed the progression of lung adenocarcinoma mainly depends on tumor cell reprogramming	(35)
	2020	LUAD	Discovered a cluster of tumor cells with high plasticity and the potential to transform into different states	(36)
	2021	LUAD	Analyzed unravel cell populations, states, and phenotypes in the spatial and ecologic evolution	(37)
Tumor Metabolic	2017	LUAD	Found a new metabolic phenotype of lung cancer and provide a theoretical framework	(38)
	2019	LUAD	Analyzed different expressed genes of single malignant cells with different metabolic phenotypes	(39)
Lung Cancer Treatment	2015	LUAD (cell line)	Revealed different expression patterns of individual cells induced by molecular targeted drug therapy resistance	(40)
	2021	LUAD	Characterized the different tumor microenvironment and provided prognostic information	(41)
Tumor Microenvironment	2017	LUAD	Analyzed the early immune cells, especially the innate immune cells and their molecular profiles	(42)
	2018	NSCLC	Showed the landscape of stroma and immune cells of NSCLC	(43)
	2018	NSCLC	Explored the heterogeneity and characteristics of T cells in TME	(44)
	2020	NSCLC	Reveals the diversity of B cells in the early stage of non-small cell lung cancer	(45)
	2021	NSCLC	Verified the enrichment of different macrophage subtypes in lung cancer	(46)
	2021	LUAD	Characterize shifts in the TME from early to advanced lung cancer	(47)

LUAD, lung cancer adenocarcinoma; NSCLC, non-small cell lung cancer; SCLC, small cell lung cancer; GGN, ground glass nodule; SSN, subsolid nodule; CNV, copy number variation.

## General Tumor Evolution Process and Unique Characteristics in the Early Stage of Lung Cancer

The development of lung cancer is a multistep process defined by spatiotemporal interactions between heterogeneous cell types, including the malignant, immune, and stromal cells in a complex ecosystem (48). The functional diversity of immune cells is especially critical for the generation of the different regulator and effector responses required to safeguard the host against cancer, while survived tumor cells evolve to actively evade immune surveillance (49). The innate and adaptive arms of our immune system act as a complementary network of self-defense against the early progression from normal to malignant (50). Despite the fact that the immune system can identify and destroy nascent tumor cells, it can also be hijacked to promote tumor initiation and progression (51, 52). The dual anti-tumor and pro-tumor roles of immunity are referred to as cancer immunoediting (50). Immunoediting consists of three processes that function to control and shape cancer development either independently or in sequence. In the elimination phase, innate and the adaptive immune systems recognize transformed cells and destroy them, resulting in a return to normal physiological tissue (53). However, if antitumor immunity fails to eliminate transformed cells (also known as immunoselection), survived tumor cells may enter into the equilibrium phase, when the adaptive immunity prevents tumor outgrowth (53). Then, these cell variants may eventually acquire further mutations that help them evade immune surveillance, and progress to clinically detectable malignancies in the escape phase (54).

**Figure 1:**
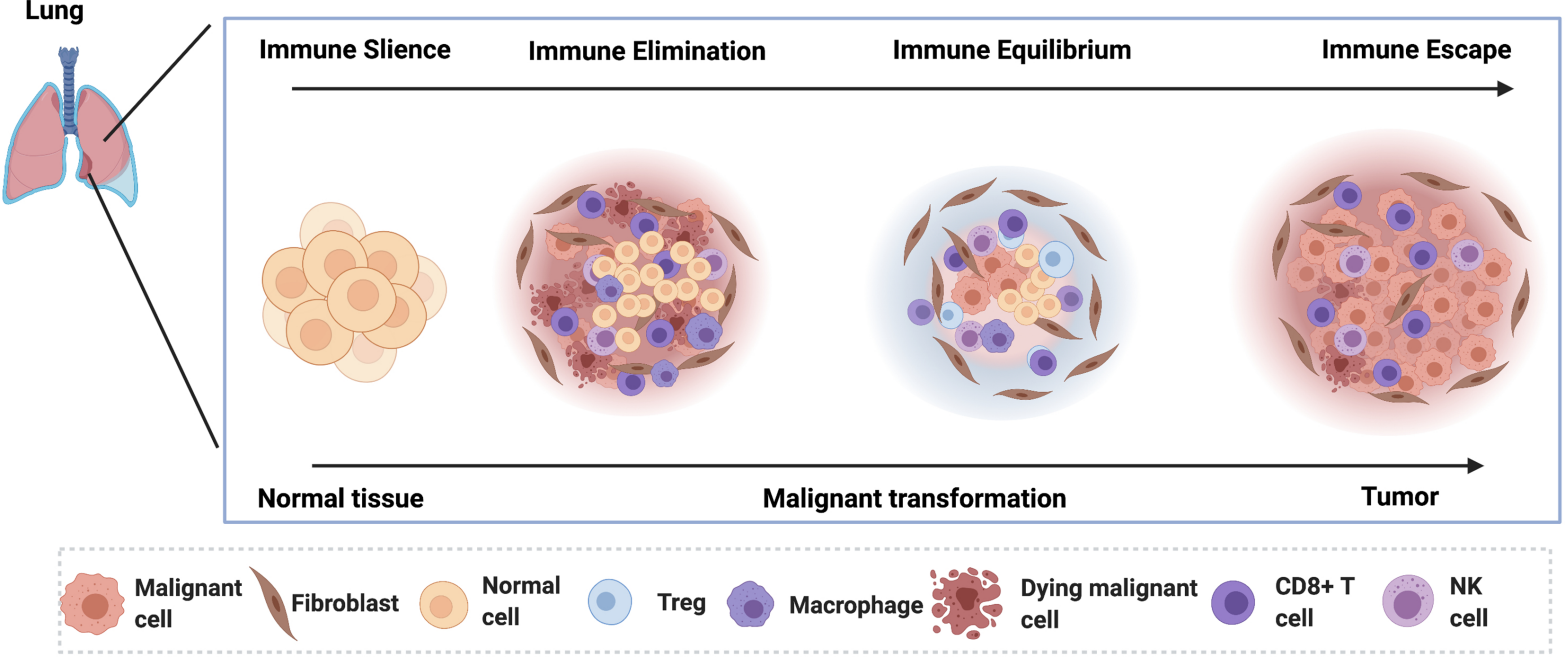
The lung cancer tumorigenesis in the early stage of lung cancer is depicted. In the pre-lesion, the immune cells dominate the microenvironment and eliminate the malignant cells by inducing the cell death. In the immune equilibrium phase, the malignant cells become quiescent under the control of the activated immune cells. As the disease progress, the malignant cells escape the immune surveillance.

The immune responses in the process of lung cancer evolution gradually transit from immune activation to immunosuppression, characterized by decreased T-cell clonotypes, increased infiltration of regulatory T cells, and the reduced infiltration of cytotoxic T cells and anti-tumor helper T cells (55). Meanwhile, the driver mutations, chromosomal copy number aberrations, and abnormal epigenetic events in tumor cells work together to influence host immune responses (56). These results reveal that the early development of lung cancer is a continuous and gradual process modulated by the immune-editing mechanism (**Figure 1**). However, it was demonstrated that the precancerous mutated cells in some early-stage lung cancer patients were already able to suppress the immune system and escape the immune surveillance before the invasive stage, which is contradictive to the supposed elimination phase of immune-editing theory (57, 58). Thus, the developmental process in the early stage of lung cancer does not have to go through the three immune-editing phases in sequence. Some early mutations can confer the tumor cells with strong immunosuppressive capability, paralyzing anti-tumor immune responses to early tumor development (59). Consequently, those tumor cells might skip the elimination and equilibrium phase and jump into the “escape” phase. Besides, the highly heterogeneous tumor immune microenvironments of individual patients also limit the wide applicability of the immune-editing theory. Furthermore, the detailed regulatory pathways that determine the phase transition during the immune-editing process remain elusive. Conventional strategies using bulk cell populations are unable to fully delineate the various cell types and states engaged in the immune process toward malignancy, impeding further the investigation and precise interventional therapy (60). By contrast, single-cell techniques can classify the individual cells, gain into the multi-dimensional interactions between the tumor and the immune system, characterize the variations in their molecular profiles and developmental processes, and then contribute to the development of novel and practical strategies for the immune diagnosis and intervention of early-stage lung cancer (61) (**Table 2**). Here, we will highlight current findings and the potential application of single-cell technology in deciphering the evolution process of early stage of lung cancer.

**Table 2:** Summary of advantages and disadvantages of current single-cell sequencing platforms in studying the evolution of early-stage LUAD.

Molecular level	mRNA		mRNA+ Protemoics	Genome	Epigenome
Method	Smart-seq2	Droplet-based scRNA-seq	CITE-seq, Mars-seq	SNS, SCI-seq	sciATAC-seq; scATAC-seq
**Indications**	Alternative splicing of genes	Differential gene expression calling	Analysis of targeted populations;phenotypic classifications based on surface protein and transcriptomic	Recording of the interaction between mutant tumor cells and the immune cells’ behaviors	Epigenetic biomarkers for early cancer diagonstic and epigentic regulation of genes
		Allelic expression of genes			
		Gene regulatory network reconstruction dynamic changes and heterogeneity cell percentage and subtypes			
				The cell clonal evolution	
		Cell trajectory inference			
**Advantages**	Full-length transrcpit to find the mutation and splicing alteration of tumor cell	Available commercial kits	Rare cell-type dicovery and more presice in cell phenotype identification	Genetic deterministic genes in governing the emergence and maintenance of heterogeneity and colonel evolution	Investigation of regulatory state transitions and chromatin- modifying proteins in malignant transformation
		High content to identify different types and heterogeneity			
		Sufficient quantity and quality of gene detections			
**Disadvantages**	Pathology misdiagnosis in early stage of lung cancer		High cost; difficult to standarized in different labs	Missing information about transcriptional heterogeneity during tumor progression	Difficult to determine how cells navigate these regulatory transitions toward malignant
	Hard to identify the lineage tracing of cell phenotypes and rare cell types				
	Hard to characterize the clonality, inter-patient ITH, and initiation tumor site				
	Require live cells and high sample quality				
**Reference**	Marjanovic, N.D., et al. (36)	(33, 43, 45, 47, 62)	Lavin, Y., et al. (35); LaFave, L.M., et al. (42); Leader, Grout et al. (62)	Rooney, Shukla et al. (63)	LaFave, L.M., et al. (35); Marjanovic, N.D., et al. (36)

NSCLC, non-small cell lung cancer; Smart-seq2, Switching Mechanism At the end of the 5’-end of the RNA Transcript; scRNA, single-cell RNA sequencing; SNS, single-nucleus sequencing; SCI-seq, single-cell combinatorial indexed sequencing; scATAC-seq, Single-cell sequencing assay for transposase- accessible chromatin; ITH, intratumor heterogeneity; CITE-seq, cellular Indexing of Transcriptomes and Epitopes by Sequencing; Mars-seq, massively parallel single-cell RNA-Seq.

## Application of Single-Cell Technologies in the Early Stage of Lung Cancer

### Single-Cell RNA Sequencing in the Early Stage of Lung Cancer

Single-cell RNA sequencing (ScRNA-seq) technologies allow the dissection of the gene expression at single-cell resolution, revolutionizing transcriptomic studies from various aspects such as cell clustering, trajectory inference, differential expression calling, alternative splicing, allelic expression, and gene regulatory network reconstruction (64). These techniques have paved the way for the discovery of previously unknown cell types and subtypes in the normal and diseased lung, especially facilitating the study of rare cells (65). Furthermore, scRNA-seq can characterize immune cells and tumor cells in an unbiased manner at the same time (66).

The analysis of the single-cell transcriptomes of the seven stages of the mouse lung tumors, from pre-neoplastic hyperplasia to adenocarcinoma, found that the diversity of transcriptional states in the tumor and immune cells increased over time (36). In human subjects, a scRNA-seq study of 16 subsolid nodules samples and 6 adjacent normal lung tissues revealed that the cytotoxic natural killer T cells were dominant in the TME of subsolid nodules, and the malignant cells in the subsolid nodules underwent strong metabolic reprogramming and immune stress (67). Besides, a scRNA-seq analysis of ground-glass nodules (GGNs) demonstrated that the proliferation of the cancer cells was inhibited, and the immune cells were more activated in the GGN, compared with the activated proliferation of the cancer cells and the suppressive immune cells in the solid adenocarcinoma (32). A study of a total of 16 subsolid nodules (SSNs) samples from 16 treatment-naive patients provided single-cell transcriptomic profiling of SSN and their TME and indicated that SSNs exhibited more indolent biological behaviors than solid LUAD, that cytotoxic natural killer/T cells dominated in the TME of SSN, that malignant cells in SSN underwent enhanced immune stress, and that the subtype composition of endothelial cells was more like that in normal lung samples in SSN (62). ScRNA-seq has unmasked the complexity and heterogeneity of tumor–immune interplay during the transformation from pre-malignant lesions to cancerous damage.

ScRNA-seq analysis also helps elucidate the detailed interactions between tumor cells and immune cells in the early stage of lung cancer. A multiscale single-cell profiling of 35 early-stage NSCLC lesions found that a key cellular module consisting of *PDCD1^+^ CXCL13* ^+^ activated T cells, IgG^+^ plasma cells, and *SPP1* ^+^ macrophages, closely associated with evasion of lung cancer cells (62). A scRNA-seq analysis of human and mouse lung tumors unveiled that tissue-resident macrophages accumulated close to tumor cells promoted epithelial–mesenchymal transition and invasiveness, and induced a potent regulatory T cell response that protected tumor cells from adaptive immunity during early tumor formation (46). Consistently, Lavin et al. also demonstrated that Treg and non-functional T cells were enriched but cytolytic natural killer cells were excluded in the early LUAD lesions (42). Furthermore, Guo, Zhang et al. showed that there was a significant proportion of inter-tissue effector T cells with a highly migratory nature and that a high ratio of “pre-exhausted” to exhausted T cells was associated with a better prognosis of lung adenocarcinoma (42, 44). In addition, a study of seven stage-I/II LUAD samples harboring *EGFR* mutations and five tumor-adjacent lung tissues revealed that the adenocarcinoma cells were characterized by activated cell proliferation and antigen presentation to immune cells (68).

By comparing malignant lung samples with the non-malignant counterparts, scRNA-seq also uncovered the different and plastic interaction patterns between immune cells and normal cells or malignant cells. Lambrechts et al. identified some heterogeneous sub-subpopulations in stromal cells and the transcription factors that regulate their heterogeneity in the early LUAD patients by scRNA-seq (43). ScRNA-seq analysis of 10 normal lung tissues and 10 fresh LUAD tissues found that the TME was composed of cancer-associated myofibroblasts, exhausted CD8^+^ T cells, proinflammatory monocyte-derived macrophages, plasmacytoid dendritic cells, myeloid dendritic cells, anti-inflammatory monocyte-derived macrophages, normal-like myofibroblasts, NK cells, and conventional T cells (69). Multi-region of five early-stage LUADs and 14 multi-region normal lung tissues found that the Treg^+^ cells are increased in normal tissues with proximity to LUAD and the signatures and fractions of cytotoxic CD8+ T cells, antigen-presenting macrophages, and inflammatory dendritic cells were decreased (37).

ScRNA also unveiled the different characteristics of tumor–immune interplays between early-stage and advanced-stage LUAD. Compared with the early-stage LUAD, scRNA-seq demonstrated the naïve-like B cells decreased in advanced NSCLC, and their lower number was associated with poor prognosis (45). Based on scRNA-seq data of 29 lung samples of different developmental stages, Chen, Huang et al. found that advanced malignant cells exhibited a remarkably more complex TME and higher intratumor heterogeneity level than early malignant cells. In terms of immune cells, the proportions of CD8^+^/cytotoxic T cells, Treg+ T cells, and follicular B cells remarkedly differed in early and advanced LUAD. Notably, the ligand-receptor analysis found that the GNAI2-DRD2 and C4B-CD46 pairs were only detected in advanced LUAD, while COL3A1-MAG, HLA-C-SLC9C2, and COL2A1-MAG were uniquely expressed in early LUAD (47).

Collectively, scRNA-seq has helped characterize the cellular phenotypes of various immune and tumor cell types and reveal their interactions within the TME during the development of lung cancer. However, there are many limitations by now. One of the major challenges is the paucity of human patient samples available for scRNA-seq in the early stage of lung cancer. Further validation by alternative methods or larger patient cohorts is required (70). Furthermore, sample quality is a big issue because it is impossible to separate the LUAD featured with ground glass nodules from solid adenocarcinoma by pathological methods when studying the initiation of LUAD (45). Moreover, the scRNA-seq lacks the power to distinguish the ground glass opacification part and the solid part in the same LUAD, which is vital to identify the initiation site of the cellular activation module. Furthermore, it is difficult to interpret the evolution process of specific cells, identify cell phenotypes for lineage tracing, acquire cell surface antigens information, characterize the intratumor clonal heterogeneity and inter-patient clonal heterogeneity, and identify genomic alterations by scRNA-seq. Therefore, we will further discuss other single-cell techniques and their potential applications in the investigation of early-stage lung cancer development.

### Single-Cell Genome Sequencing in the Early Stage of Lung Cancer

In the initiation of lung cancer, a single normal cell gradually evolves into a malignant tumor cell and forms distinct subpopulations, which then lead to intratumoral heterogeneity and clonal diversity by genomic alterations (71). Copy number variations or single-nucleotide variations in *EGFR, RBM10*, *MET, BRAF, K-Ras*, and *TP53* were found to be functionally important in the evolution of lung cancer (72). The genome doubling and ongoing dynamic chromosomal instability in *CDK4, FOXA1*, and *BCL11A* also resulted in the progression of lung cancer (73, 74). These genomic alterations are also present in early-stage lung cancer cells, determining their sensitivity to the immune cells and associating with immune cells’ phenotypes (75). For example, patients harboring *KRAS* mutations displayed significantly lower levels of dysfunctional immune T-cell markers: PD-1 and TIM-3, in the tumors than those with wild-type *KRAS*, which indicated a suppressive immune microenvironment in *EGFR-*mutated tumors (76). Furthermore, it was found that the *ADCY8*, *PIK3CA*, and *CDKN2A* mutations were associated with remarkedly decreased expression of the immune-inhibitory ligand: PD-L1 (77), which indicated an upregulated immune response in early-stage lung cancer patients with these gene mutations. McGranahan et al. demonstrated the positive association of high tumor mutation burdens with more activated CD8^+^ T cells and higher levels of PD-L1 expression in early-stage NSCLC (78).

Although these results revealed the important roles of gene mutations in the early stage of lung cancer by interacting with the immune system, most of the results derived from the sequencing data of bulk tumor cells, which is difficult to unmask the deeper underlying genotypic and phenotypic heterogeneity that exists inter- and intra-tumors. Furthermore, the number of cells harboring the mutations and the zygosity of these mutations cannot be accurately assessed by bulk genome sequencing (79). Moreover, current findings were insufficient to clarify the interactions between the individual mutant tumor cells and the immune cells when the normal epithelial cells transformed to malignant cells (63). Meanwhile, it remains unclear how the components of adaptive immune system individually respond to the transformation at the genome level (80). Single-cell DNA sequencing may overcome these obstacles by detecting the founder mutations and sub-clonal mutations in tumor cells at a single-cell level (81). Further application of the newly developed single-genome technique, such as SNS-seq (single-nucleus sequencing), LIANT (single-cell whole-genome analyses by linear amplification *via* transposon insertion), and SCI-seq (single-cell combinatorial indexed sequencing) (82) may unravel the clonal relationship between different malignant cells and dissect the immune responses contributed by the genomic elements of the individual cells during the progression from pre-malignant lesion to advanced oncogenesis (83).

### Single-Cell Epigenome Sequencing in the Early Stage of Lung Cancer

Cellular heterogeneity of individual cells within the tumor–immune ecosystem is displayed not only in the genome and transcriptome, but also in the epigenome. Epigenetic alterations, including DNA methylation, histone modifications, and non-coding RNA expression, have been reported to play an important role in the tumorigenesis of lung cancer (84, 85). At the epigenetic level, the histone H3 lysine 36 methyltransferase NSD3 could promote the development of lung squamous cell carcinoma (86). The DNA methyltransferase inhibitors and histone deacetylase inhibitors could reverse tumor immune evasion in NSCLC by modulating the T-cell exhaustion state towards the memory and effector T-cell phenotypes (87, 88). Besides, the antigen presentations of the immune cells are also altered by epigenetic modulations with the hypomethylating agents or histone deacetylase inhibitors (89). These findings demonstrated the vital roles of epigenetic regulation in cancer evolution. However, the detailed mechanisms of how these epigenetic events modulate the immunoediting process in specific cell types remain unclear during lung cancer early development.

Single-cell epigenome profiling methods include scATAC-seq (assay for transposase-accessible chromatin in single cells with sequencing), scCHIP-seq (single-cell chromatin immune-precipitation followed by sequencing), sciHi-C (single-cell combinatorial indexed Hi-C), and scCUT&Tag (single-cell cleavage under targets & tagmentation) (90–93). ScATAC-seq can reveal the chromatin accessibility landscape that governs the transcriptional regulation in different cell populations (94). Sn-m3C-seq (single-nucleus methyl-3C sequencing) can give information about chromatin organization and DNA methylation and distinguish the heterogeneous cell types (95). Smart-RRBS (single-cell methylome and transcriptome analysis) can detect the methylation status in the promoters of specific tumor suppressor genes and the overall number of hypermethylated genes, which increases with the neoplastic progression from hyperplasia to adenocarcinoma (96). A scATAC-seq analysis of the K-Ras+/LSLG12D;p53frt/frt (KP) mouse model found that the cancer cells were tightly regulated by the smarca4, which regulated the activity of the lung lineage SWI/SNF transcription factor and ultimately accelerated tumor progression (35). Using the KP mice and sciATAC-seq (combinatorial indexing to identify single cells without single-cell isolation for chromatin accessibility), LaFave, Kartha et al. also defined co-accessible regulatory programs and inferred key activating and repressive chromatin regulators of epigenetic changes in the tumor cells, including RUNX transcription factors (which are predictive biomarkers for the survival of LUAD patients) (35).

Together, these results demonstrated the power of single-cell epigenomics to identify regulatory programs and key biomarkers during tumor progression. Combined single-cell methods have also emerged to allow analyses of epigenetic–transcriptional correlations, thereby enabling detailed investigations of how epigenetic states modulate cell phenotypes and the immune editing process.

### Single-Cell Proteomics in the Early Stage of Lung Cancer

Although single-cell transcriptomic, genomic, and epigenomic methods have been informative about gene expression and genome landscapes and have demonstrated vital basic research and clinical value in lung cancer, information on proteins is also important and necessary since proteins are the cellular workhorses (97). Methods of protein detection at the single-cell level include flow cytometry, Sc-MS (liquid chromatography mass spectrometry-based single-cell proteomics), ScoPE (isobaric labeling for single-cell proteomics), CyTOF (cytometry by time-of-flight), SCITO-seq (single-cell combinatorial indexed cytometry sequencing), CITE-seq (cellular indexing of transcriptomes and epitopes by sequencing), Mars-seq (massively parallel single-cell RNA-Seq), and SCPFC (single-cell phospho-specific flow cytometry) (98–100). Some of these methods can simultaneously measure multiple cellular proteins and RNA at the single-cell level.

Leader Grout et al. applied CITE-seq combining phenotypic classifications based on surface protein expression and transcriptomic profile, to characterize the cellular classification and increase our understanding of the immune cellular landscape in the mouse model of early-stage lung cancer (62). Lin et al. utilized SCPFC in the investigation of signaling network interactions and unraveled the dynamic changes of tyrosine phospho-Stat1 (pStat1) in lung cancer cells in a mouse model (101). More recently, Rahman et al. performed a CyTOF analysis of cell suspensions derived from tissues of early-stage LUAD after surgical resection. They found that high levels of cerium were specifically associated with a phenotypically distinct subset of lung macrophages that were most prevalent in noninvolved lung tissue, whereas tumor-associated macrophages had lower levels of cerium (99). The CyTOF combined with mars-seq2 fully characterized the immune landscape of early-stage LUAD and distinguished the immune changes driven by the tumor lesion from those driven by the lung tissue (42).

In sum, single-cell proteomics can add another dimension to clarify the substantial heterogeneity and complicated interaction among seemingly identical cells at the genome level, significantly contributing to the quantitative understanding of the developmental mechanisms of early-stage lung cancer.

### Single-Cell Metabolic Profiling in the Early Stage of Lung Cancer

The metabolic reprogramming is fundamental to both cancer cells and responding immune cells during cancer development (102, 103). Moreover, metabolic heterogeneity and plasticity exist in diverse cells, especially in the immune cells responding to cancer cells (104–106). A recent finding indicated that lactate acid secreted by the glycolytic cancer cells favored the activation of the immune cells toward an immunosuppressive phenotype (107). In addition, the cancer cells could harness the metabolic by-products to induce the immune suppressive microenvironment (108).

The single-cell metabolomics field is at its very early stage at this moment. The sc-MS (single-cell metabolic profiling by mass cytometry) (109, 110) and the SCENITH (Flow Cytometry-Based Method to Functionally Profile Energy Metabolism with Single-Cell Resolution) were recently developed (111). These technologies could reveal global metabolic functions and determine complex and linked immune phenotypes in rare cell subpopulations (112, 113).

### Lineage Tracing Combined With Single-Cell Sequencing in the Early Stage of Lung Cancer

Another long-standing quest is to understand the developmental origin and the cell fate determination of each cell within a tissue (114). Cell states are highly flexible and present multipotent characteristics before reaching differentiation destination. A comprehensive study of the molecular alterations during cell fate determination would be useful to better clarify those steps involved in the precancerous stage of lung tumor. Using the methods of lineage tracing with single-cell technology such as CellTaging (a combinatorial cell indexing approach), TracerSeq (transposon-based barcoding sequencing), scGESTALT (single-cell genome editing of synthetic target arrays for lineage tracing), and MEMOIR (memory by engineered mutagenesis with optical *in situ* readout), we can investigate an individual cell early and track the states of its clonal progeny at a later time point *via* sequencing of the inherited DNA sequences, or “barcodes” (115–117). These methods offer an opportunity to integrate complementary information about both cell lineage and cell states into synthetical views of cell a differentiation destination and dynamic interactions between the tumor and immune cells (118, 119). ScRNA-seq combined with lineage tracing allows simultaneous measurement of cell identity and developmental origin at single-cell resolution (119). Zepp et al. revealed that the transformation from the alveolar type 1 progenitor cells to alveolar type 2 cells in the mesenchymal alveolar niche of the lung is important for the tissue injury response by combining scRNA-seq and signaling lineage reporter system (58, 120). Furthermore, Wellenstein et al. tracked the fate determination process of immune cells in response to the antigens expressed specifically on the surface of nearby tumor cells during the immune editing process (121). Labeling cell subpopulations across the lung region by the Dre-Rox or Cre-LoxP recombination system together with single-cell sequencing technology has potential to be used for simultaneously investigating the reciprocal evolution of tumor cells and immune cells as well as their fate determinations in the lesion at the initial stage (122, 123).

Together, parallel advances in single-cell sequencing techniques and lineage tracing methods facilitate the mapping of the clonal relationships onto the tumor immune landscape and help decipher the crosstalk between the tumor and immune cells during the whole developmental process.

### Single-Cell Spatial Omics in the Early Stage of Lung Cancer

Recent advances in spatially resolved methods allow us to achieve transcriptional cell-type classifications, map cellular spatial distributions in tissues, and reveal the intracellular and intercellular networks in lung tumors (124). Genome sequencing analyses of 25 spatially distinct regions of early-stage NSCLC found that the driver mutations displayed sub-clonal diversification in different regions, embodying the value of combining spatial information with sequencing data in deciphering the mechanism of the evolution of lung cancer cells (125). Many single-cell spatial transcriptomics combines spatial barcoding-based methods (ST, Visium, HDST, Slidesee, Naostring, GeoMx, DBiT-seq, and Zipcode) and imaging-based methods (osm-FISH, MERFISH, SeqFish, STARMAP, and FISSEQ) (126–128). These technologies may deepen our understanding of the functional organization of the tissue and the cellular and molecular mechanism on how cancer cells modify their surroundings to generate an immune suppressive microenvironment in the early-stage lung cancer (126–128). Indeed, single-cell spatial transcriptomics has already started to be used to delineate the precise landscape of the TME and the crosstalk between the tumor and immune cells at both cellular and sub-cellular levels (129). A spatial transcriptomics analysis of LUAD and LUSC samples has demonstrated the spatial gene expression atlas and spatial heterogeneity variation between LUAD and LUSC as well as differences in normal and cancerous regions (130).

The immune responses occurring in the early stage of lung cancer are mediated by not only the cell–cell interaction, but also the coordinated actions of a diverse set of cytokines (131). In the early stage of lung cancer, the majority of the cytokines consists of IFN-γ, IL-12, and TNF-α, whereas the concentration of the pro-angiogenic cytokine VEGF is extremely low (132, 133). These cytokines are crucial for regulating the immune equilibrium. However, the spatial localization of tissue-resident immune or tumor cells producing specific modulatory cytokines remains elusive. The application of the spatial genomic sequencing method at the single-cell level can specifically identify the signaling interactions and communications between the immune and tumor cells in the early stage of lung cancer (115, 127). Together, analyzing single-cell gene expression in a spatially resolved context is critical for understanding the heterotypic interactions among the cells in the TME in the early stage of lung cancer.

## Conclusion and Perspective

**Figure 2:**
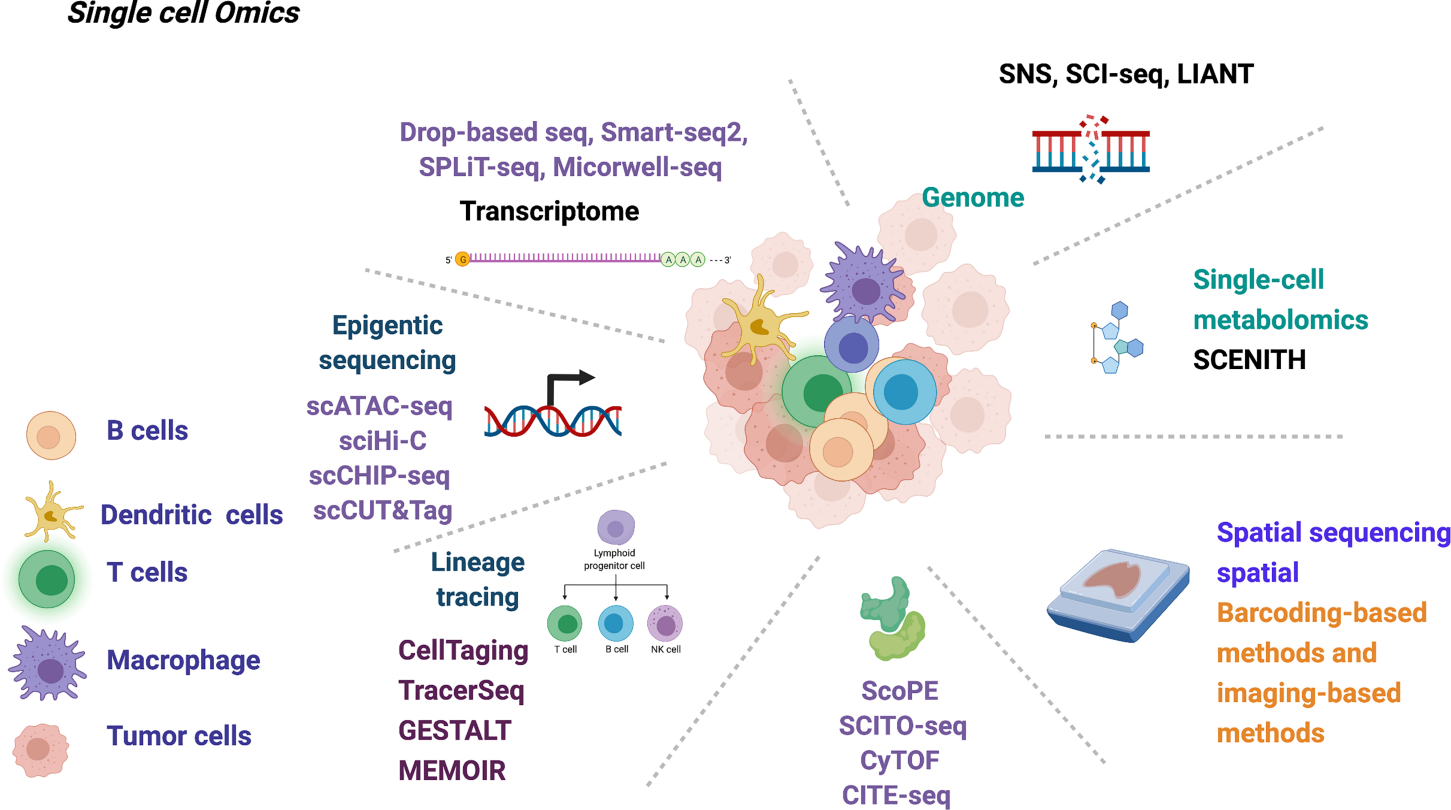
Summary of current single-cell multi-omics technology that may be used in deciphering the early-stage lung cancer evolution.

A comprehensive understanding of the tumor immune microenvironment is vital to treatment options and prognosis of lung cancer. Multi-omics simultaneous profiling of gene expression, genetic variation, epigenetic change, cell surface proteins, metabolic activities, and spatial information from the same single cell allows full and robust delineation of the developmental plasticity and immune-mediated pruning of the tumor cells from multiple dimensions during the early development of lung cancer (**Figure 2**).

The goal of tumor immunology research is to be able to manipulate the immune cells/molecules to prevent and treat cancer (134). A deeper understanding of the immune–tumor interplay during early-stage lung cancer by single-cell sequencing technology can help identify novel immunotherapy targets, determine which patients may benefit most from immunotherapy, and discover new mechanisms of resistance to immunotherapy (135).

In sum, our views of the applications of the multi-omics single-cell techniques in the early stage of lung cancer will contribute to broadening their application in relevant basic research and boosting the development of immunotherapy for early-stage lung cancer.

## Author Contributions

All authors critically wrote and revised the manuscript. All authors contributed to the article and approved the submitted version.

## Funding

The research was supported by the National Key R&D Program of China (2019YFA0906100 and 2020YFA0804100); the National Natural Science Foundation of China (32170919 and 92042305); Shanghai Pulmonary Hospital Innovation Team (FKCX1906); Shanghai Science and Technology Committee (20YF1441100, 20XD1403000, 18DZ2293400); Shenzhen Science and Technology Program (KQTD2016113015442590) and Natural Science Foundation of Guangdong (2021A1515010919).

## Conflict of Interest

The authors declare that the research was conducted in the absence of any commercial or financial relationships that could be construed as a potential conflict of interest.

## Publisher’s Note

All claims expressed in this article are solely those of the authors and do not necessarily represent those of their affiliated organizations, or those of the publisher, the editors and the reviewers. Any product that may be evaluated in this article, or claim that may be made by its manufacturer, is not guaranteed or endorsed by the publisher.

## References

[1] Siegel RL Miller KD Jemal A. Cancer Statistics, 2020. CA Cancer J Clin (2020) 70(1):7–30. doi: 10.3322/caac.21590 31912902

[2] Wei W Zeng H Zheng R Zhang S An L Chen R. Cancer Registration in China and its Role in Cancer Prevention and Control. Lancet Oncol (2020) 21(7):e342–9. doi: 10.1016/S1470-2045(20)30073-5 32615118

[3] Owada-Ozaki Y Muto S Takagi H Inoue T Watanabe Y Fukuhara M. Prognostic Impact of Tumor Mutation Burden in Patients With Completely Resected Non-Small Cell Lung Cancer: Brief Report. J Thorac Oncol (2018) 13(8):1217–21. doi: 10.1016/j.jtho.2018.04.003 29654927

[4] Tavernari D Battistello E Dheilly E Petruzzella AS Mina M Sordet-Dessimoz J. Non-Genetic Evolution Drives Lung Adenocarcinoma Spatial Heterogeneity and Progression. Cancer Discov (2021) 11(6):1490–507. doi: 10.1158/2159-8290.CD-20-1274 33563664

[5] Sharma A Merritt E Hu X Cruz A Jiang C Sarkodie H. Non-Genetic Intra-Tumor Heterogeneity Is a Major Predictor of Phenotypic Heterogeneity and Ongoing Evolutionary Dynamics in Lung Tumors. Cell Rep (2019) 29(8):2164–2174 e5. doi: 10.1016/j.celrep.2019.10.045 31747591 PMC6952742

[6] Detterbeck FC Boffa DJ Kim AW Tanoue LT. The Eighth Edition Lung Cancer Stage Classification. Chest (2017) 151(1):193–203. doi: 10.1016/j.chest.2016.10.010 27780786

[7] Gettinger S Horn L Jackman D Spigel D Antonia S Hellmann M. Five-Year Follow-Up of Nivolumab in Previously Treated Advanced Non-Small-Cell Lung Cancer: Results From the CA209-003 Study. J Clin Oncol (2018) 36(17):1675–84. doi: 10.1200/JCO.2017.77.0412 29570421

[8] Manser R Wright G Hart D Byrnes G Campbell DA. Surgery for Early Stage Non-Small Cell Lung Cancer. Cochrane Database Syst Rev (2005) 2005(1):Cd004699. doi: 10.1002/14651858.CD004699.pub2 PMC8407335 15674959

[9] Blandin Knight S Crosbie PA Balata H Chudziak J Hussell T Dive C. Progress and Prospects of Early Detection in Lung Cancer. Open Biol (2017) 7(9):170070. doi: 10.1098/rsob.170070 28878044 PMC5627048

[10] Liu C Li H Xu K Song S He Y Cai X. Multiple Primary Lung Cancer Versus Intrapulmonary Metastatic Cancer: A Case of Multiple Pulmonary Nodules. Thorac Cancer (2019) 10(2):352–8. doi: 10.1111/1759-7714.12918 PMC6360236 30548923

[11] Riley RS June CH Langer R Mitchell MJ. Delivery Technologies for Cancer Immunotherapy. Nat Rev Drug Discovery (2019) 18(3):175–96. doi: 10.1038/s41573-018-0006-z PMC6410566 30622344

[12] Yang Y. Cancer Immunotherapy: Harnessing the Immune System to Battle Cancer. J Clin Invest (2015) 125(9):3335–7. doi: 10.1172/JCI83871 PMC4588312 26325031

[13] Ko EC Raben D Formenti SC. The Integration of Radiotherapy With Immunotherapy for the Treatment of Non-Small Cell Lung Cancer. Clin Cancer Res (2018) 24(23):5792–806. doi: 10.1158/1078-0432.CCR-17-3620 29945993

[14] Rosner S Reuss JE Forde PM. PD-1 Blockade in Early-Stage Lung Cancer. Annu Rev Med (2019) 70:425–35. doi: 10.1146/annurev-med-050217-025205 30355264

[15] Liu SY Wu YL. Ongoing Clinical Trials of PD-1 and PD-L1 Inhibitors for Lung Cancer in China. J Hematol Oncol (2017) 10(1):136. doi: 10.1186/s13045-017-0506-z 28679395 PMC5499002

[16] Forde PM Chaft JE Smith KN Anagnostou V Cottrell TR Hellmann MD. Neoadjuvant PD-1 Blockade in Resectable Lung Cancer. N Engl J Med (2018) 378(21):1976–86. doi: 10.1056/NEJMoa1716078 PMC6223617 29658848

[17] Hoy H Lynch T Beck M. Surgical Treatment of Lung Cancer. Crit Care Nurs Clin North Am (2019) 31(3):303–13. doi: 10.1016/j.cnc.2019.05.002 31351552

[18] Ghysen K Vansteenkiste J. Immunotherapy in Patients With Early Stage Resectable Nonsmall Cell Lung Cancer. Curr Opin Oncol (2019) 31(1):13–7. doi: 10.1097/CCO.0000000000000497 30325753

[19] Walk EE Yohe SL Beckman A Schade A Zutter MM Pfeifer J. The Cancer Immunotherapy Biomarker Testing Landscape. Arch Pathol Lab Med (2020) 144(6):706–24. doi: 10.5858/arpa.2018-0584-CP 31714809

[20] Gonzalez H Hagerling C Werb Z. Roles of the Immune System in Cancer: From Tumor Initiation to Metastatic Progression. Genes Dev (2018) 32(19-20):1267–84. doi: 10.1101/gad.314617.118 PMC6169832 30275043

[21] O’Donnell JS Teng MWL Smyth MJ. Cancer Immunoediting and Resistance to T Cell-Based Immunotherapy. Nat Rev Clin Oncol (2019) 16(3):151–67. doi: 10.1038/s41571-018-0142-8 30523282

[22] Bruni D Angell HK Galon J. The Immune Contexture and Immunoscore in Cancer Prognosis and Therapeutic Efficacy. Nat Rev Cancer (2020) 20(11):662–80. doi: 10.1038/s41568-020-0285-7 32753728

[23] Zhang Y Liu F. Multidimensional Single-Cell Analyses in Organ Development and Maintenance. Trends Cell Biol (2019) 29(6):477–86. doi: 10.1016/j.tcb.2019.02.006 30928527

[24] Wu F Fan J He Y Xiong A Yu J Li Y. Single-Cell Profiling of Tumor Heterogeneity and the Microenvironment in Advanced Non-Small Cell Lung Cancer. Nat Commun (2021) 12(1):2540. doi: 10.1038/s41467-021-22801-0 33953163 PMC8100173

[25] Baslan T Hicks J. Unravelling Biology and Shifting Paradigms in Cancer With Single-Cell Sequencing. Nat Rev Cancer (2017) 17(9):557–69. doi: 10.1038/nrc.2017.58 28835719

[26] Gawad C Koh W Quake SR. Single-Cell Genome Sequencing: Current State of the Science. Nat Rev Genet (2016) 17(3):175–88. doi: 10.1038/nrg.2015.16 26806412

[27] Hedlund E Deng Q. Single-Cell RNA Sequencing: Technical Advancements and Biological Applications. Mol Aspects Med (2018) 59:36–46. doi: 10.1016/j.mam.2017.07.003 28754496

[28] Greenberg AK Yee H Rom WN. Preneoplastic Lesions of the Lung. Respir Res (2002) 3(1):20–0. doi: 10.1186/rr170 PMC107849 11980589

[29] Klebe S Henderson DW. Facts and Fiction: Premalignant Lesions of Lung Tissues. Pathology (2013) 45(3):305–15. doi: 10.1097/PAT.0b013e32835f45fd 23448809

[30] Min JW Kim WJ Han JA Jung YJ Kim KT Park WY. Identification of Distinct Tumor Subpopulations in Lung Adenocarcinoma *via* Single-Cell RNA-Seq. PloS One (2015) 10(8):e0135817. doi: 10.1371/journal.pone.0135817 26305796 PMC4549254

[31] Xiong D Pan J Yin Y Jiang H Szabo E Lubet RA. Novel Mutational Landscapes and Expression Signatures of Lung Squamous Cell Carcinoma. Oncotarget (2018) 9(7):7424–41. doi: 10.18632/oncotarget.23716 PMC5800913 29484121

[32] Lu T Yang X Shi Y Zhao M Bi G Liang J. Single-Cell Transcriptome Atlas of Lung Adenocarcinoma Featured With Ground Glass Nodules. Cell Discov (2020) 6:69. doi: 10.1038/s41421-020-00200-x 33083004 PMC7536439

[33] Xing X Yang F Huang Q Guo H Li J Qiu M. Decoding the Multicellular Ecosystem of Lung Adenocarcinoma Manifested as Pulmonary Subsolid Nodules by Single-Cell RNA Sequencing. Sci Adv (2021) 7(5):eabd9738. doi: 10.1126/sciadv.abd9738 33571124 PMC7840134

[34] Rothwell DG Li Y Ayub M Tate C Newton G Hey Y. Evaluation and Validation of a Robust Single Cell RNA-Amplification Protocol Through Transcriptional Profiling of Enriched Lung Cancer Initiating Cells. BMC Genomics (2014) 15:1129. doi: 10.1186/1471-2164-15-1129 25519510 PMC4320548

[35] LaFave LM Kartha VK Ma S Meli K Del Priore I Lareau C. Epigenomic State Transitions Characterize Tumor Progression in Mouse Lung Adenocarcinoma. Cancer Cell (2020) 38(2):212–.e13. doi: 10.1016/j.ccell.2020.06.006 32707078 PMC7641015

[36] Marjanovic ND Hofree M Chan JE Canner D Wu K Trakala M. Emergence of a High-Plasticity Cell State During Lung Cancer Evolution. Cancer Cell (2020) 38(2):229–46.e13. doi: 10.1016/j.ccell.2020.06.012 32707077 PMC7745838

[37] Sinjab A Han G Treekitkarnmongkol W Hara K Brennan PM Dang M. Resolving the Spatial and Cellular Architecture of Lung Adenocarcinoma by Multiregion Single-Cell Sequencing. Cancer Discov (2021) 11(10):2506–23. doi: 10.1158/2159-8290.CD-20-1285 PMC8487926 33972311

[38] Yu L Lu M Jia D Ma J Ben-Jacob E Levine H. Modeling the Genetic Regulation of Cancer Metabolism: Interplay Between Glycolysis and Oxidative Phosphorylation. Cancer Res (2017) 77(7):1564–74. doi: 10.1158/0008-5472.CAN-16-2074 PMC5380541 28202516

[39] Li Z Wang Z Tang Y Lu X Chen J Dong Y. Liquid Biopsy-Based Single-Cell Metabolic Phenotyping of Lung Cancer Patients for Informative Diagnostics. Nat Commun (2019) 10(1):3856. doi: 10.1038/s41467-019-11808-3 31451693 PMC6710267

[40] Suzuki A Matsushima K Makinoshima H Sugano S Kohno T Tsuchihara K. Single-Cell Analysis of Lung Adenocarcinoma Cell Lines Reveals Diverse Expression Patterns of Individual Cells Invoked by a Molecular Target Drug Treatment. Genome Biol (2015) 16:66. doi: 10.1186/s13059-015-0636-y 25887790 PMC4450998

[41] Kim M Min YK Jang J Park H Lee S Lee CH. Single-Cell RNA Sequencing Reveals Distinct Cellular Factors for Response to Immunotherapy Targeting CD73 and PD-1 in Colorectal Cancer. J Immunother Cancer (2021) 9(7):e002503. doi: 10.1136/jitc-2021-002503 34253638 PMC8276303

[42] Lavin Y Kobayashi S Leader A Amir E-AD Elefant N Bigenwald C. Innate Immune Landscape in Early Lung Adenocarcinoma by Paired Single-Cell Analyses. Cell (2017) 169(4):750–65.e17. doi: 10.1016/j.cell.2017.04.014 28475900 PMC5737939

[43] Lambrechts D Wauters E Boeckx B Aibar S Nittner D Burton O. Phenotype Molding of Stromal Cells in the Lung Tumor Microenvironment. Nat Med (2018) 24(8):1277–89. doi: 10.1038/s41591-018-0096-5 29988129

[44] Guo X Zhang Y Zheng L Zheng C Song J Zhang Q. Global Characterization of T Cells in Non-Small-Cell Lung Cancer by Single-Cell Sequencing. Nat Med (2018) 24(7):978–85. doi: 10.1038/s41591-018-0045-3 29942094

[45] Chen J Tan Y Sun F Hou L Zhang C Ge T. Single-Cell Transcriptome and Antigen-Immunoglobin Analysis Reveals the Diversity of B Cells in Non-Small Cell Lung Cancer. Genome Biol (2020) 21(1):152. doi: 10.1186/s13059-020-02064-6 32580738 PMC7315523

[46] Casanova-Acebes M Dalla E Leader AM LeBerichel J Nikolic J Morales BM. Tissue-Resident Macrophages Provide a Pro-Tumorigenic Niche to Early NSCLC Cells. Nature (2021) 595(7868):578–84. doi: 10.1038/s41586-021-03651-8 PMC8923521 34135508

[47] Chen Z Huang Y Hu Z Zhao M Li M Bi G. Landscape and Dynamics of Single Tumor and Immune Cells in Early and Advanced-Stage Lung Adenocarcinoma. Clin Transl Med (2021) 11(3):e350. doi: 10.1002/ctm2.350 33783985 PMC7943914

[48] Nasim F Sabath BF Eapen GA. Lung Cancer. Med Clin North Am (2019) 103(3):463–73. doi: 10.1016/j.mcna.2018.12.006 30955514

[49] Schreiber RD Old LJ Smyth MJ. Cancer Immunoediting: Integrating Immunity’s Roles in Cancer Suppression and Promotion. Science (2011) 331(6024):1565–70. doi: 10.1126/science.1203486 21436444

[50] Rosenthal R Cadieux EL Salgado R Bakir MA Moore DA Hiley CT. Neoantigen-Directed Immune Escape in Lung Cancer Evolution. Nature (2019) 567(7749):479–85. doi: 10.1038/s41586-019-1032-7 PMC6954100 30894752

[51] Vesely MD Kershaw MH Schreiber RD Smyth MJ. Natural Innate and Adaptive Immunity to Cancer. Annu Rev Immunol (2011) 29:235–71. doi: 10.1146/annurev-immunol-031210-101324 21219185

[52] Dunn GP Old LJ Schreiber RD. The Immunobiology of Cancer Immunosurveillance and Immunoediting. Immunity (2004) 21(2):137–48. doi: 10.1016/j.immuni.2004.07.017 15308095

[53] Dunn GP Bruce AT Ikeda H Old LJ Schreiber RD. Cancer Immunoediting: From Immunosurveillance to Tumor Escape. Nat Immunol (2002) 3(11):991–8. doi: 10.1038/ni1102-991 12407406

[54] Kunimasa K Goto T. Immunosurveillance and Immunoediting of Lung Cancer: Current Perspectives and Challenges. Int J Mol Sci (2020) 21(2):597. doi: 10.3390/ijms21020597 PMC7014343 31963413

[55] Saab S Zalzale H Rahal Z Khalifeh Y Sinjab A Kadara H. Insights Into Lung Cancer Immune-Based Biology, Prevention, and Treatment. Front Immunol (2020) 11(159). doi: 10.3389/fimmu.2020.00159 PMC7026250 32117295

[56] Dejima H Hu X Chen R Zhang J Zhang J. Immune Evolution From Preneoplasia to Invasive Lung Adenocarcinomas and Underlying Molecular Features. Nat Commun (2021) 12(1):2722. doi: 10.1038/s41467-021-22890-x 33976164 PMC8113327

[57] Singhal S Stadanlick J Annunziata MJ Rao AS Bhojnagarwala PS O’Brien S. Human Tumor-Associated Monocytes/Macrophages and Their Regulation of T Cell Responses in Early-Stage Lung Cancer. Sci Transl Med (2019) 11(479):eaat1500. doi: 10.1126/scitranslmed.aat1500 30760579 PMC6800123

[58] Vinay DS Ryan EP Pawelec G Talib WH Stagg J Elkord E. Immune Evasion in Cancer: Mechanistic Basis and Therapeutic Strategies. Semin Cancer Biol (2015) 35(Suppl):S185–98. doi: 10.1016/j.semcancer.2015.03.004 25818339

[59] Zitvogel L Tesniere A Kroemer G. Cancer Despite Immunosurveillance: Immunoselection and Immunosubversion. Nat Rev Immunol (2006) 6(10):715–27. doi: 10.1038/nri1936 16977338

[60] Wiel C Le Gal K Ibrahim MX Jahangir CA Kashif M Yao H. BACH1 Stabilization by Antioxidants Stimulates Lung Cancer Metastasis. Cell (2019) 178(2):330–45.e22. doi: 10.1016/j.cell.2019.06.005 31257027

[61] Seijo LM Peled N Ajona D Boeri M Field JK Sozzi G. Biomarkers in Lung Cancer Screening: Achievements, Promises, and Challenges. J Thorac Oncol (2019) 14(3):343–57. doi: 10.1016/j.jtho.2018.11.023 PMC6494979 30529598

[62] Leader AM Grout JA Chang C Maier B Tabachnikova A Walker L. CITEseq Analysis of Non-Small-Cell Lung Cancer Lesions Reveals an Axis of Immune Cell Activation Associated With Tumor Antigen Load and TP53 Mutations. Cold Spring Harbor Laboratory (2020).

[63] Rooney MS Shukla SA Wu CJ Getz G Hacohen N. Molecular and Genetic Properties of Tumors Associated With Local Immune Cytolytic Activity. Cell (2015) 160(1-2):48–61. doi: 10.1016/j.cell.2014.12.033 25594174 PMC4856474

[64] Chen G Ning B Shi T. Single-Cell RNA-Seq Technologies and Related Computational Data Analysis. Front Genet (2019) 10:317. doi: 10.3389/fgene.2019.00317 31024627 PMC6460256

[65] Schiller HB Montoro DT Simon LM Rawlins EL Meyer KB Strunz M. The Human Lung Cell Atlas: A High-Resolution Reference Map of the Human Lung in Health and Disease. Am J Respir Cell Mol Biol (2019) 61(1):31–41. doi: 10.1165/rcmb.2018-0416TR 30995076 PMC6604220

[66] Papalexi E Satija R. Single-Cell RNA Sequencing to Explore Immune Cell Heterogeneity. Nat Rev Immunol (2018) 18(1):35–45. doi: 10.1038/nri.2017.76 28787399

[67] Reyfman PA Walter JM Joshi N Anekalla KR McQuattie-Pimentel AC Chiu S. Single-Cell Transcriptomic Analysis of Human Lung Provides Insights Into the Pathobiology of Pulmonary Fibrosis. Am J Respir Crit Care Med (2019) 199(12):1517–36. doi: 10.1164/rccm.201712-2410OC PMC6580683 30554520

[68] He D Wang D Lu P Yang N Xue Z Zhu X. Single-Cell RNA Sequencing Reveals Heterogeneous Tumor and Immune Cell Populations in Early-Stage Lung Adenocarcinomas Harboring EGFR Mutations. Oncogene (2021) 40(2):355–68. doi: 10.1038/s41388-020-01528-0 PMC7808940 33144684

[69] Bischoff P Trinks A Obermayer B Pett JP Lehmann A Jurmeister P. Single-Cell RNA Sequencing Reveals Distinct Tumor Microenvironmental Patterns in Lung Adenocarcinoma. Oncogene (2021). doi: 10.1038/s41388-021-02054-3 PMC8677623 34663877

[70] Batson J Royer L Webber J. Molecular Cross-Validation for Single-Cell RNA-Seq. bioRxiv (2019) 786269. doi: 10.1101/786269

[71] McGranahan N Swanton C. Clonal Heterogeneity and Tumor Evolution: Past, Present, and the Future. Cell (2017) 168(4):613–28. doi: 10.1016/j.cell.2017.01.018 28187284

[72] Reiniger L Téglási V Pipek O Rojkó L Glasz T Vágvölgyi A. Tumor Necrosis Correlates With PD-L1 and PD-1 Expression in Lung Adenocarcinoma. Acta Oncol (2019) 58(8):1087–94. doi: 10.1080/0284186X.2019.1598575 31002007

[73] Kerr EM Gaude E Turrell FK Frezza C Martins CP. Mutant Kras Copy Number Defines Metabolic Reprogramming and Therapeutic Susceptibilities. Nature (2016) 531(7592):110–3. doi: 10.1038/nature16967 PMC4780242 26909577

[74] Ciabatti S Cammelli S Frakulli R Arcelli A Macchia G Deodato F. Radiotherapy of Pancreatic Cancer in Older Patients: A Systematic Review. J Geriatr Oncol (2019) 10(4):534–9. doi: 10.1016/j.jgo.2018.09.007 30270196

[75] Schumacher TN Schreiber RD. Neoantigens in Cancer Immunotherapy. Science (2015) 348(6230):69–74. doi: 10.1126/science.aaa4971 25838375

[76] Datar I Sanmamed MF Wang J Henick BS Choi J Badri T. Expression Analysis and Significance of PD-1, LAG-3, and TIM-3 in Human Non–Small Cell Lung Cancer Using Spatially Resolved and Multiparametric Single-Cell Analysis. Clin Cancer Res (2019) 25(15):4663–73. doi: 10.1158/1078-0432.CCR-18-4142 PMC7444693 31053602

[77] Choi M Kadara H Zhang J Parra ER Rodriguez-Canales J Gaffney SG. Mutation Profiles in Early-Stage Lung Squamous Cell Carcinoma With Clinical Follow-Up and Correlation With Markers of Immune Function. Ann Oncol (2017) 28(1):83–9. doi: 10.1093/annonc/mdw437 PMC6246501 28177435

[78] McGranahan N Furness AJS Rosenthal R Ramskov S Lyngaa R Saini SK. Clonal Neoantigens Elicit T Cell Immunoreactivity and Sensitivity to Immune Checkpoint Blockade. Science (2016) 351(6280):1463–9. doi: 10.1126/science.aaf1490 PMC4984254 26940869

[79] Shi X Chakraborty P Chaudhuri A. Unmasking Tumor Heterogeneity and Clonal Evolution by Single-Cell Analysis. J Cancer Metastasis Treat (2018) 4:47. doi: 10.20517/2394-4722.2018.32

[80] Turajlic S Sottoriva A Graham T Swanton C. Resolving Genetic Heterogeneity in Cancer. Nat Rev Genet (2019) 20(7):404–16. doi: 10.1038/s41576-019-0114-6 30918367

[81] Rizvi NA Hellmann MD Snyder A Kvistborg P Makarov V Havel JJ. Mutational Landscape Determines Sensitivity to PD-1 Blockade in Non–Small Cell Lung Cancer. Science (2015) 348(6230):124–8. doi: 10.1126/science.aaa1348 PMC4993154 25765070

[82] Leung ML Davis A Gao R Casasent A Wang Y Sei E. Single-Cell DNA Sequencing Reveals a Late-Dissemination Model in Metastatic Colorectal Cancer. Genome Res (2017) 27(8):1287–99. doi: 10.1101/gr.209973.116 PMC5538546 28546418

[83] Baslan T Kendall J Rodgers L Cox H Riggs M Stepansky A. Genome-Wide Copy Number Analysis of Single Cells. Nat Protoc (2012) 7(6):1024–41. doi: 10.1038/nprot.2012.039 PMC5069701 22555242

[84] Darılmaz Yüce G Ortaç Ersoy E. [Lung Cancer and Epigenetic Modifications]. Tuberk Toraks (2016) 64(2):163–70. doi: 10.5578/tt.10231 27481083

[85] Duruisseaux M Esteller M. Lung Cancer Epigenetics: From Knowledge to Applications. Semin Cancer Biol (2018) 51:116–28. doi: 10.1016/j.semcancer.2017.09.005 28919484

[86] Yuan G Flores NM Hausmann S Lofgren SM Kharchenko V Angulo-Ibanez M. Elevated NSD3 Histone Methylation Activity Drives Squamous Cell Lung Cancer. Nature (2021) 590(7846):504–8. doi: 10.1038/s41586-020-03170-y PMC7895461 33536620

[87] Mehta A Dobersch S Romero-Olmedo AJ Barreto G. Epigenetics in Lung Cancer Diagnosis and Therapy. Cancer Metastasis Rev (2015) 34(2):229–41. doi: 10.1007/s10555-015-9563-3 25939322

[88] Franco F Jaccard A Romero P Yu YR Ho PC. Metabolic and Epigenetic Regulation of T-Cell Exhaustion. Nat Metab (2020) 2(10):1001–12. doi: 10.1038/s42255-020-00280-9 32958939

[89] Kommalapati A Tanvetyanon T. Epigenetic Modulation of Immunotherapy Cofactors to Enhance Tumor Response in Lung Cancer. Hum Vaccines Immunotherapeut (2021) 17(1):51–4. doi: 10.1080/21645515.2020.1764273 PMC7872084 32460615

[90] Cui Z Cui Y Gao Y Jiang T Zang T Wang Y. Enhancement and Imputation of Peak Signal Enables Accurate Cell-Type Classification in scATAC-Seq. Front Genet (2021) 12:658352. doi: 10.3389/fgene.2021.658352 33889181 PMC8056015

[91] Ku WL Nakamura K Gao W Cui K Hu G Tang Q. Single-Cell Chromatin Immunocleavage Sequencing (Scchic-Seq) to Profile Histone Modification. Nat Methods (2019) 16(4):323–5. doi: 10.1038/s41592-019-0361-7 PMC7187538 30923384

[92] Ramani V Deng X Qiu R Gunderson KL Steemers FJ Disteche CM. Massively Multiplex Single-Cell Hi-C. Nat Methods (2017) 14(3):263–6. doi: 10.1038/nmeth.4155 PMC5330809 28135255

[93] Bartosovic M Kabbe M Castelo-Branco G. Single-Cell CUT&Tag Profiles Histone Modifications and Transcription Factors in Complex Tissues. Nat Biotechnol (2021) 39(7):825–35. doi: 10.1038/s41587-021-00869-9 PMC7611252 33846645

[94] Buenrostro JD Wu B Litzenburger UM Ruff D Gonzales ML Snyder MP. Single-Cell Chromatin Accessibility Reveals Principles of Regulatory Variation. Nature (2015) 523(7561):486–90. doi: 10.1038/nature14590 PMC4685948 26083756

[95] Lee D-S Luo C Zhou J Chandran S Rivkin A Bartlett A. Simultaneous Profiling of 3D Genome Structure and DNA Methylation in Single Human Cells. Nat Methods (2019) 16(10):999–1006. doi: 10.1038/s41592-019-0547-z 31501549 PMC6765423

[96] Gu H Raman AT Wang X Gaiti F Chaligne R Mohammad AW. Smart-RRBS for Single-Cell Methylome and Transcriptome Analysis. Nat Protoc (2021) 16(8):4004–30. doi: 10.1038/s41596-021-00571-9 PMC8672372 34244697

[97] Nigro E Imperlini E Scudiero O Monaco ML Polito R Mazzarella G. Differentially Expressed and Activated Proteins Associated With Non Small Cell Lung Cancer Tissues. Respir Res (2015) 16(1):74. doi: 10.1186/s12931-015-0234-2 26104294 PMC4487583

[98] Kelly RT. Single-Cell Proteomics: Progress and Prospects. Mol Cell Proteomics (2020) 19(11):1739–48. doi: 10.1074/mcp.R120.002234 PMC7664119 32847821

[99] Rahman AH Lavin Y Kobayashi S Leader A Merad M. High-Dimensional Single Cell Mapping of Cerium Distribution in the Lung Immune Microenvironment of an Active Smoker. Cytomet B Clin Cytom (2018) 94(6):941–5. doi: 10.1002/cyto.b.21545 PMC6289862 28734132

[100] Dou M Clair G Tsai CF Xu K Chrisler WB Sontag RL. High-Throughput Single Cell Proteomics Enabled by Multiplex Isobaric Labeling in a Nanodroplet Sample Preparation Platform. Anal Chem (2019) 91(20):13119–27. doi: 10.1021/acs.analchem.9b03349 PMC7192326 31509397

[101] Lin CC Huang WL Su WP Chen HH Lai WW Yan JJ. Single Cell Phospho-Specific Flow Cytometry can Detect Dynamic Changes of Phospho-Stat1 Level in Lung Cancer Cells. Cytomet A (2010) 77(11):1008–19. doi: 10.1002/cyto.a.20965 20814891

[102] O’Neill LAJ Pearce EJ. Immunometabolism Governs Dendritic Cell and Macrophage Function. J Exp Med (2016) 213(1):15–23. doi: 10.1084/jem.20151570 26694970 PMC4710204

[103] Weber GF. Metabolism in Cancer Metastasis. Int J Cancer (2016) 138(9):2061–6. doi: 10.1002/ijc.29839 26355498

[104] Schmidt DR Patel R Kirsch DG Lewis CA Vander Heiden MG Locasale JW. Metabolomics in Cancer Research and Emerging Applications in Clinical Oncology. CA: A Cancer J Clin (2021) 71(4):333–58. doi: 10.3322/caac.21670 PMC8298088 33982817

[105] Bergers G Fendt S-M. The Metabolism of Cancer Cells During Metastasis. Nat Rev Cancer (2021) 21:162–80. doi: 10.1038/s41568-020-00320-2 PMC8733955 33462499

[106] Leone RD Powell JD. Metabolism of Immune Cells in Cancer. Nat Rev Cancer (2020) 20(9):516–31. doi: 10.1038/s41568-020-0273-y PMC8041116 32632251

[107] Huang H Zhou P Wei J Long L Shi H Dhungana Y. *In Vivo* CRISPR Screening Reveals Nutrient Signaling Processes Underpinning CD8+ T Cell Fate Decisions. Cell (2021) 184(5):1245–61.e21. doi: 10.1016/j.cell.2021.02.021 PMC8101447 33636132

[108] Zappasodi R Serganova I Cohen IJ Maeda M Shindo M Senbabaoglu Y. CTLA-4 Blockade Drives Loss of Treg Stability in Glycolysis-Low Tumours. Nature (2021) 591(7851):652–8. doi: 10.1038/s41586-021-03326-4 PMC8057670 33588426

[109] Shrestha B. Ten Major Future Challenges in Single-Cell Metabolomics, in Single Cell Metabolism: Methods and Protocols. Shrestha B, editor. New York, NY: Springer New York (2020) p. 219–23. 10.1007/978-1-4939-9831-9_16 31565777

[110] Evers TMJ Hochane M Tans SJ Heeren RMA Semrau S Nemes P. Deciphering Metabolic Heterogeneity by Single-Cell Analysis. Analytical Chem (2019) 91(21):13314–23. doi: 10.1021/acs.analchem.9b02410 PMC6922888 31549807

[111] Arguello RJ Combes AJ Char R Gigan JP Baaziz AI Bousiquot E. SCENITH: A Flow Cytometry-Based Method to Functionally Profile Energy Metabolism With Single-Cell Resolution. Cell Metab (2020) 32(6):1063–.e7. doi: 10.1016/j.cmet.2020.11.007 33264598 PMC8407169

[112] Vodnala SK Eil R Kishton RJ Sukumar M Yamamoto TN Ha NH. T Cell Stemness and Dysfunction in Tumors Are Triggered by a Common Mechanism. Science (2019) 363(6434):eaau0135. doi: 10.1126/science.aau0135 30923193 PMC8194369

[113] Depeaux K Delgoffe GM. Metabolic Barriers to Cancer Immunotherapy. Nat Rev Immunol (2021) 21:785–97. doi: 10.1038/s41577-021-00541-y PMC8553800 33927375

[114] Sheng N Li Y Qian R Li Y. The Clinical Significance and Biological Function of lncRNA RGMB-AS1 in Hepatocellular Carcinoma. BioMed Pharmacother (2018) 98:577–84. doi: 10.1016/j.biopha.2017.12.067 29288973

[115] Hu KH Eichorst JP McGinnis CS Patterson DM Chow ED Kersten K. ZipSeq: Barcoding for Real-Time Mapping of Single Cell Transcriptomes. Nat Methods (2020) 17(8):833–43. doi: 10.1101/2020.02.04.932988 PMC7891292 32632238

[116] Wu SS Lee JH Koo BK. Lineage Tracing: Computational Reconstruction Goes Beyond the Limit of Imaging. Mol Cells (2019) 42(2):104–12. doi: 10.14348/molcells.2019.0006 PMC6399003 30764600

[117] Wagner DE Weinreb C Collins ZM Briggs JA Megason SG Klein AM. Single-Cell Mapping of Gene Expression Landscapes and Lineage in the Zebrafish Embryo. Science (2018) 360(6392):981–7. doi: 10.1126/science.aar4362 PMC6083445 29700229

[118] Figueres-Oñate M Sánchez-Villalón M Sánchez-González R López-Mascaraque L. Lineage Tracing and Cell Potential of Postnatal Single Progenitor Cells *In Vivo* . Stem Cell Rep (2019) 13(4):700–12. doi: 10.1016/j.stemcr.2019.08.010 PMC6829765 31543472

[119] Praktiknjo SD Obermayer B Zhu Q Fang L Liu H Quinn H. Tracing Tumorigenesis in a Solid Tumor Model at Single-Cell Resolution. Nat Commun (2020) 11(1):991. doi: 10.1038/s41467-020-14777-0 32080185 PMC7033116

[120] Zepp JA Zacharias WJ Frank DB Cavanaugh CA Zhou S Morley MP. Distinct Mesenchymal Lineages and Niches Promote Epithelial Self-Renewal and Myofibrogenesis in the Lung. Cell (2017) 170(6):1134–48.e10. doi: 10.1016/j.cell.2017.07.034 28886382 PMC5718193

[121] Wellenstein MD de Visser KE. Cancer-Cell-Intrinsic Mechanisms Shaping the Tumor Immune Landscape. Immunity (2018) 48(3):399–416. doi: 10.1016/j.immuni.2018.03.004 29562192

[122] Wagner DE Klein AM. Lineage Tracing Meets Single-Cell Omics: Opportunities and Challenges. Nat Rev Genet (2020) 21(7):410–27. doi: 10.1038/s41576-020-0223-2 PMC7307462 32235876

[123] Liu Q Liu K Cui G Huang X Yao S Guo W. Lung Regeneration by Multipotent Stem Cells Residing at the Bronchioalveolar-Duct Junction. Nat Genet (2019) 51(4):728–38. doi: 10.1038/s41588-019-0346-6 30778223

[124] Lein E Borm LE Linnarsson S. The Promise of Spatial Transcriptomics for Neuroscience in the Era of Molecular Cell Typing. Science (2017) 358(6359):64–9. doi: 10.1126/science.aan6827 28983044

[125] De Bruin EC McGranahan N Mitter R Salm M Wedge DC Yates L. Spatial and Temporal Diversity in Genomic Instability Processes Defines Lung Cancer Evolution. Science (2014) 346(6206):251–6. doi: 10.1126/science.1253462 PMC4636050 25301630

[126] Moor AE Itzkovitz S. Spatial Transcriptomics: Paving the Way for Tissue-Level Systems Biology. Curr Opin Biotechnol (2017) 46:126–33. doi: 10.1016/j.copbio.2017.02.004 28346891

[127] Baccin C Al-Sabah J Velten L Helbling PM Grünschläger F Hernández-Malmierca P. Combined Single-Cell and Spatial Transcriptomics Reveal the Molecular, Cellular and Spatial Bone Marrow Niche Organization. Nat Cell Biol (2020) 22(1):38–48. doi: 10.1038/s41556-019-0439-6 31871321 PMC7610809

[128] Wang X Allen WE Wright MA Sylwestrak EL Samusik N Vesuna S. Three-Dimensional Intact-Tissue Sequencing of Single-Cell Transcriptional States. Science (2018) 361(6400):eaat5691. doi: 10.1126/science.aat5691 29930089 PMC6339868

[129] Kumar MP Du J Lagoudas G Jiao Y Sawyer A Drummond DC. Analysis of Single-Cell RNA-Seq Identifies Cell-Cell Communication Associated With Tumor Characteristics. Cell Rep (2018) 25(6):1458–68.e4. doi: 10.1016/j.celrep.2018.10.047 30404002 PMC7009724

[130] Zhang L Mao S Yao M Chao N Yang Y Ni Y. Spatial transcriptome sequencing revealed spatial trajectory in the Non-Small Cell Lung Carcinoma. bioRxiv (2021) 2021.04.26.441394. doi: 10.1101/2021.04.26.441394

[131] Mascaux C Angelova M Vasaturo A Beane J Hijazi K Anthoine G. Immune Evasion Before Tumour Invasion in Early Lung Squamous Carcinogenesis. Nature (2019) 571(7766):570–5. doi: 10.1038/s41586-019-1330-0 31243362

[132] Meng L Jiang X Liang J Pan Y Pan F Liu D. Postoperative Psychological Stress and Expression of Stress-Related Factors HSP70 and IFN-γ in Patients With Early Lung Cancer. Minerva Med (2020). doi: 10.23736/S0026-4806.20.06658-6 32538589

[133] Eruslanov EB Bhojnagarwala PS Quatromoni JG Stephen TL Ranganathan A Deshpande C. Tumor-Associated Neutrophils Stimulate T Cell Responses in Early-Stage Human Lung Cancer. J Clin Invest (2014) 124(12):5466–80. doi: 10.1172/JCI77053 PMC4348966 25384214

[134] Laughney AM Hu J Campbell NR Bakhoum SF Setty M Lavallee VP. Regenerative Lineages and Immune-Mediated Pruning in Lung Cancer Metastasis. Nat Med (2020) 26(2):259–69. doi: 10.1038/s41591-019-0750-6 PMC7021003 32042191

[135] Beaumont KG Beaumont MA Sebra R. Application of Single-Cell Sequencing to Immunotherapy. Urol Clin North Am (2020) 47(4):475–85. doi: 10.1016/j.ucl.2020.07.005 33008498

